# Mechanical Treatment of Some Skin Anomalies*Presented at Meeting of the State Medical Association, April, 1893.

**Published:** 1893-06

**Authors:** M. B. Hutchins

**Affiliations:** Atlanta, Ga.; Lecturer on Diseases of the Skin Atlanta Medical College; 330 Equitable Building


### Southern Medical Record.

A MONTHLY JOURNAL OF MEDICINE AND SURGERY.

VOL. XXIII. ATLANTA, GA., JUNE, 1893. NO. 6.

### Original Articles.

### MECHANICAL TREATMENT OF SOME SKIN ANOMALIES.*

BY M. B. HUTCHINS, M. D., ATLANTA, GA.

Lecturer on Diseases of the Skin Atlanta Medical College.

This paper will treat of some of the various mechanical means of removing anomalies of the skin. As the text-books go generally into the subject, I shall, for the most part, limit my remarks to my own practical experience.

The destruction of superfluous hairs, of moles, "birth marks," and the various simple growths of the skin will be described. Malignant diseases and "tumors" do not properly come within the scope of this paper.

About the year 1879 Dr. Michel, of St. Louis, successfully employed electrolysis for the destruction of the hairs in trichiasis. Dr. Hardaway, a dermatologist of the same city, followed the idea in the treatment of hypertrichosis. Various dermatologists have since reported upon its successful use. Hair upon the lip and face of women affords the usual field of operation. Three to eight cells of a galvanic battery are used. For the past two years I have used some "Law" cells put together by myself containing as the fluid an aqueous solution of sal

*Presented at Meeting of the State Medical Association, April, 1893.

ammoniac. For the destruction of hairs five of these are sufficient, though they have not been filled in two years. Seven of these cells suffice for ordinary moles. To the negative pole a pencil shaped needle holder containing a number 8 or 12 ordinary sewing needle is attached, while the positive pole is provided with the usual sponge electrode. Expensive needles, etc. can be dispensed with. The needle is attached to the negative pole for the reason that electrolysis through the positive will leave a black dot at the point of insertion of the needle.

I prefer the needle-holder having a spring for opening or closing the circuit as this leaves the entire control of the current to me, the patient steadily holding the well-wetted sponge electrode in one hand instead of having to open and close the circuit by letting go or catching hold of the sponge.

The needle is carefully inserted into the hair follicle, parallel to the root, until slight resistance, the bottom of the follicle and the seat of the hair papilla, is felt. The circuit is then closed and the current allowed to pass until a frothy substance the size of a small pin head fills the follicle and a small area of blanching appears around the needle. If the needle has followed the course of the hair root and remained in the follicle the pain is less than when the root sheaths are pierced, and the frothy substance is more abundant. These are practical points worth remembering. Now, if the hair is easily pulled out you may be quite sure there will be no re-growth, but if it pulls out with difficulty, or breaks off, the current must immediately be re-applied. A pair of tweezers, but preferably a pair of forceps made for the purpose, are used to extract the hairs.

The pain is very like a bee-sting after the circuit is completed, but patients not only get used to it a kind of local anæsthesia soon occurs. The mere insertion of the needle, in any of these operations, seems to give no pain. I have frequently found that patients who were unable, or unwilling, to stand the removal of more than ten or fifteen hairs at the first sitting would soon endure the removal of as many as seventy-five in an hour. Frequent wetting of the operated surface with ether cools the burning sensation which often occurs, or a lotion of alcohol, water and oxide of zinc answers. The heat and redness do not last long anyway. There is slight danger of producing enough destructive action and inflammation to leave a little scarring if the hairs are removed side by side and in numbers at a sitting, but I have frequently disregarded this in the case of patients from a distance who had but little time for the work, and with no bad results. Unnecessary strength of the current is likely to have too much of a solvent action and leave marks. Of course those hairs only can be satisfactorily treated whose follicies can be seen, a lucky circumstance for the physician, as, otherwise, his time and patience would be exhausted with lanugo hairs, to say nothing if his eye-sight.

It is possible that the estimate of ten to twenty-five cent. of re-growths is too high, and there should be no per cent. if the operator is sure to destroy the hair papilla, and if the hair comes out with no resistance. The natural growth of the fine, lanugo hairs as time passes and the possible stimulation by the current acting in their vicinity are to be allowed for. Moles in which the treated hairs grow tend to decrease under the current, and hairs in moles treated directly soon become dead looking and cease to grow, but the best results are obtained from treating each directly.

Illustrating the treatment of hypertrichosis, I may mention the case of a lady of forty-three from whose chin and face I removed about three hundred hairs in a period of two and a half years, and about the same number from the upper lip. One begins on these cases with a very limited idea of the number of hairs to be removed and you may expect your estimate to be about seventy-five per cent. too low, and in a patient of this age you may expect to find the lanugo hairs becoming full grown in the intervals between treatments. This patient had a tendency to keloid and there was the danger that any little scar produced might take on this condition. Nothing like a scar was noticed until towards the last, when a few minute, whitish points were seen to mark the places at which some of the hairs had been removed, from the chin, where the current had perhaps been a little too strong. The moles treated directly had either become almost invisible or had disappeared entirely, while those which had received the current only as needed to remove the hairs had much decreased in size. The growth of hair is often made more vigorous by the pulling out or cutting off which their owners subject them to.

From the chin and face of a lady of twenty-two I removed sixty-three hairs and from the upper lip three hundred and five, in one week. There remained a fine, downy growth upon each cheek which may give trouble in the future. No bad effect followed the energetic treatment in this case. I may mention in passing that the lady wrote, some months later, to ask what she could use to decolorize a few of the hairs which showed dark in certain lights. Undiluted peroxide of hydrogen was advised and the result was satisfactory. Mention of the other cases treated would be in the nature of repetition and is therefore unnecessary. Hairs which re-grew were frequently little, black, ill-nourished stubs which were easily destroyed and which would probably have ceased to exist anyhow. The best criterion of results is the patient's opinion, they expect more than the physician—often—and if they are satisfied you may well be.

I suppose I have treated by electrolysis almost every kind of mole that exists; the flesh-colored, flat or rounded, the brownish of all shapes, and even a few small, soft black ones of the kind so closely related to those which frequently form the starting point of a sarcoma; one mole which was sore and "tender" and looked like a red berry, and two, in different patients, of skin color, tough, hard and fibrous. Six to seven cells of my battery sufficed. As in the destruction of hairs, the needle is attached to the negative pole. The smallest were transfixed, in a line parellel to the skin, nearly through the base, and the current allowed to pass until the mole became blanched and frothing occurred around the needle. If the mole were large the current was again passed, at an angle to the first puncture, and this done a third time in the very large, tough ones. In a week or ten days the small ones will have practically disappeared and the large ones markedly decreased in size. The greatest decline is seen along the line of puncture of the needle, the growth often having a lobulated appearance from this depression. At the point of puncture a small crust forms, sero-sanguinous in character, persisting a few days, then falling without leaving a mark. At times the tops of the smaller moles treated become necrotic and separate. The decrease in size of the moles goes on for some weeks, but the larger and tougher ones will not entirely disappear without repetition of the operation once to a number of times. When we get the natural result, though, we will often be unable to locate the point where the mole was situated. I have never feared setting up a malignant process in these growths and have seen none of the commonly feared effects of "tampering with moles."

A young lady who was anxious to be rid of numerous moles on her face and neck submitted to the removal of three with the curved scissors, one of the wounds being closed with a fine stitch, and to the treatment of several others by electrolysis—all at one sitting. Being "in society" and anxious for a good result, I would have been informed if she were dissatisfied, but I have no reason to believe that she was disappointed. When the mole is small and superficial it is nicely removable with curved scissors, leaving a faint, white, scarcely perceptible scar which is a vast improvement upon the appearance of the original lesion. A mole which had steadily grown since its companion above, on the face of a gentleman of 63, was treated with a quack "paste," was cut away with scissors, and the wound, having a spongy floor, was cauterized with the nitrate of silver stick. Some months later I was informed that there was a slight tendency to regrow.

Warts tend to spontaneous disappearance—hence the numerous successful "charms" for their removal. I remove the large, unsightly ones with the knife, stitching the edges of the wound together with fine sutures. The smaller ones can be no better treated than by the scissors, any possible remains being destroyed with nitrate of silver or nitric acid. Any small growth can be removed with the curette, but this is a rather harsh instrument.

On the face of a lady of 33 there were three deep purple lesions answering to what is known as the "port-wine" mark. One of these—in the center of the left cheek—had only been present eight years, the other two were congenital. The one in the cheek was the size of the finger nail, level with the skin, of a solid purple color. It was destroyed with nitric acid and as soon as the wound was in the proper state of granulation enough bran-sized grafts to cover about half the whole area were taken from the arm to the depth of the superficial capillaries and closely applied to the granulations—all under antisepsis. Rubber tissue, wet in bichloride solution, was then fitted on, then a pad of cotton, held with adhesive strips, and finally the edges of the dressing were sealed with ichthyol-collodion. At the end of four days all the grafts had nicely "taken." The final result was a smooth skin scarcely distinguishable from that surrounding it, no remains of the "mark."

A granuloma of uncertain origin upon the carmine border of the lower lip of a lady recently confined. She was 34 years old—her mother died of tuberculosis when this patient was a nursing infant. The lesion was said to have resembled a "blood blister" at first. After being "picked" it grew rapidly to pea size, became papular, soft and bled easily. The "picking" had been done by the patient with a needle. The growth was cut out with curved scissors, bled freely, was cauterized with nitrate of silver. Beginning re-growth in the front was destroyed with the same caustic; spontaneous subsidence of a second attempt at re-growth. It is two years since the operation and there has been no return of the growth.

Most of the lesions of acne vulgaris rapidly subside after puncture and free bleeding, with pressing out the contents where possible. If there is infection when you puncture, a pustule will follow. The indolent, small lesions of acne punctata are easily removable by "knocking off" their tops with the curette. The large indurate masses of diseased sebaceous glands seen about the neck and beneath the jaws in acne, are best treated by incision and thorough scraping out with the curette. This will succeed where swabbing the cavity with pure carbolic acid will fail. Antisepsis must not be neglected.

A lima-bean-sized, cavernous naevus of deep purplish color, in the front margin of the hair of an infant, was lightly touched three times with the button point of the Paquelin cautery at a red heat. A seared, brownish look with coagulation of the contained blood resulted, but the effect was only temporary. Later, the small Paquelin point was thrust into the cavity, through the skin, and thoroughly "bored around," apparently completely destroying the lesion and cutting off the blood supply. A severe open wound was left which healed slowly, and I have no definite knowledge of the result. These naevi are very hard to effectually destroy. I believe a better operation would be complete excision of the lesion, controlling the hemorrhage with the red hot cautery point, and, at the proper time, either getting the wound edges together or grafting the surface.

A congenital, capillary naevus level with the skin, at birth looking like a thumb print on the cheek of a child now four years old, has been treated with electrolysis at greater or less intervals. Formerly a dull red color, becoming livid under excitement or exposure to cold. Now the site is marked by a faint, smooth scar with a scant, fine net-work of minute red vessels. The needle, attached to the negative pole, was inserted in a vessel, or a small cluster of them, the sponge electrode being pressed to the hand or temple. From four to seven cells were used, according to their condition, the current being continued until frothing occurred and coagulation took place in the vessel treated, as well as sometimes in those immediately around. The positive pole gives a firmer clot than the negative, hence it has been advised to reverse the current for a second, when using the negative pole. The battery lately used has no reversing attachment, and it seems to me that the results obtained have been equally as good as if the current had been reversed. There can be no doubt that this treatment of the case mentioned will finally destroy every vessel and that the faint scar now present will practically disappear with the growth of the child. Owing to the use of chloroform anæsthesia the intervals between the operations have necessarily been long.

In cases of long-standing redness of the face, with dilated venules and partial stasis of blood, electrolysis affords satisfactory results. In treating dilated vessels of the skin we get both a coagulation of the blood and something of a solvent action in the tissues. A similar action takes place in treating other growths but is not so well seen. As in other cases, we get here a small crust at the site of puncture, which, falling, leaves nothing to mark its place, unless the current has been too strong, or the punctures too close together.

The superficial use of the cautery or of scarification with a fine-edged knife will destroy these vessels, but the first would leave more or less scarring and both are rather frightful to the patient or his friends. The larger vessels are sometimes treated by slitting them longitudinally with a knife, but it is a delicate and rather uncertain procedure.

I have seen a small epithelioma which was treated by electrolysis grow under the stimulation, and finally have to be excised as was first advised. Where possible it is best to thoroughly excise a beginning epthelioma, or any growth which is subject to enough irritatiou to render it liable to become malignant.

In conclusion, it is only necessary to add that the treatment, of whatever kind, employed, for an anomaly of the skin of any character, must be thorough and complete. Electrolysis is best for the destruction of hairs, of the larger moles and for dilated vessels. The tedium of the treatment is its chief draw back. Other anomalies should receive the ordinary surgical treatment. There are further plans of treating the various skin troubles mechanically but the length of this paper renders it necessary to omit mentioning them at this time.

330 Equitable Building.

---

WE call the attention of our readers to the attractive and distinctive Antikamnia advertisement in this number. This firm gladly sends samples free to physicians who will furnish their address.

